# Fludarabine as a cost-effective adjuvant to enhance engraftment of human normal and malignant hematopoiesis in immunodeficient mice

**DOI:** 10.1038/s41598-018-27425-x

**Published:** 2018-06-14

**Authors:** A. Pievani, I. M. Michelozzi, B. Rambaldi, V. Granata, A. Corsi, F. Dazzi, A. Biondi, M. Serafini

**Affiliations:** 1 M. Tettamanti Research Center, Department of Pediatrics, University of Milano-Bicocca, Monza, 20900 Italy; 2 Department of Haemato-Oncology, Rayne Institute, King’s College London, London, SE59NU UK; 3 Department of Molecular Medicine, Sapienza University of Rome, Rome, 00161 Italy

## Abstract

There is still an unmet need for xenotransplantation models that efficiently recapitulate normal and malignant human hematopoiesis. Indeed, there are a number of strategies to generate humanized mice and specific protocols, including techniques to optimize the cytokine environment of recipient mice and drug alternatives or complementary to the standard conditioning regimens, that can be significantly modulated. Unfortunately, the high costs related to the use of sophisticated mouse models may limit the application of these models to studies that require an extensive experimental design. Here, using an affordable and convenient method, we demonstrate that the administration of fludarabine (Fludara^TM^) promotes the extensive and rapid engraftment of human normal hematopoiesis in immunodeficient mice. Quantification of human CD45^+^ cells in bone marrow revealed approximately a 10^2^-fold increase in mice conditioned with irradiation plus fludarabine. Engrafted cells in the bone marrow included hematopoietic stem cells, as well as myeloid and lymphoid cells. Moreover, this model proved to be sufficient for robust reconstitution of malignant myeloid hematopoiesis, permitting primary acute myeloid leukemia cells to engraft as early as 8 weeks after the transplant. Overall, these results present a novel and affordable model for engraftment of human normal and malignant hematopoiesis in immunodeficient mice.

## Introduction

In the 2000s, various immunodeficient models were developed by combining the IL-2rg^null^ gene with conventional Prkdc^scid^ and Rag1/2^null^ mutations. These strains showed high levels of engraftment and differentiation of human hematopoietic progenitor cells, leading to remarkable advances in the development of human disease models^1^. Nevertheless, humanized mouse models are still under development, and various protocols have been established to improve human cell engraftment, in terms of rate, endurance, and function. Techniques to achieve higher levels of human cell engraftment at earlier time points include the identification of: 1) optimal sources of stem cells, 2) route of donor cell administration, 3) methods to modulate the cytokine environment of recipient mice, and 4) drug alternatives or complementary to the standard conditioning regimens. Furthermore, the identification of the factors responsible for a better engraftment of malignant human hematopoiesis, in particular, acute myeloid leukemia (AML) samples derived from patients, would be highly desirable to improve the recapitulation of the disease^2^.

In recent years, fludarabine has been used as a single agent or in combination with other drugs in the conditioning regimen before allogeneic stem cell transplantation^3–7^. This nucleoside analogue is also well known for its immunosuppressive properties, independently of its incorporation into DNA, which results in leuko- and lymphopenia in patients^8^. Notably, it has been shown that the fludarabine-induced immunosuppression is associated with the inhibition of the cytokine-induced activation of STAT1 and STAT1-dependent gene transcription in normal resting or activated lymphocytes^9^. Fludarabine could have also a role within the bone/marrow microenvironment since it has been demonstrated that this drug significantly increases bone formation in a heterotopic ossification model and promotes osteoclastogenesis^10,11^.

Despite numerous clinical studies in human hematopoietic stem cell transplantation, there are inadequate studies on the cytotoxic activity of fludarabine in a limited number of animal models. Within the context of bone marrow (BM) transplantation, fludarabine has been mainly administered in graft-versus-host disease mouse models^12–14^.

In our study, we have investigated whether the addition of fludarabine to irradiation in the conditioning regimen of a xenotransplantation mouse model would make recipients more permissive for the engraftment of normal and malignant human cells.

## Results

### Fludarabine enables efficient human cell reconstitution

**Figure 1:**
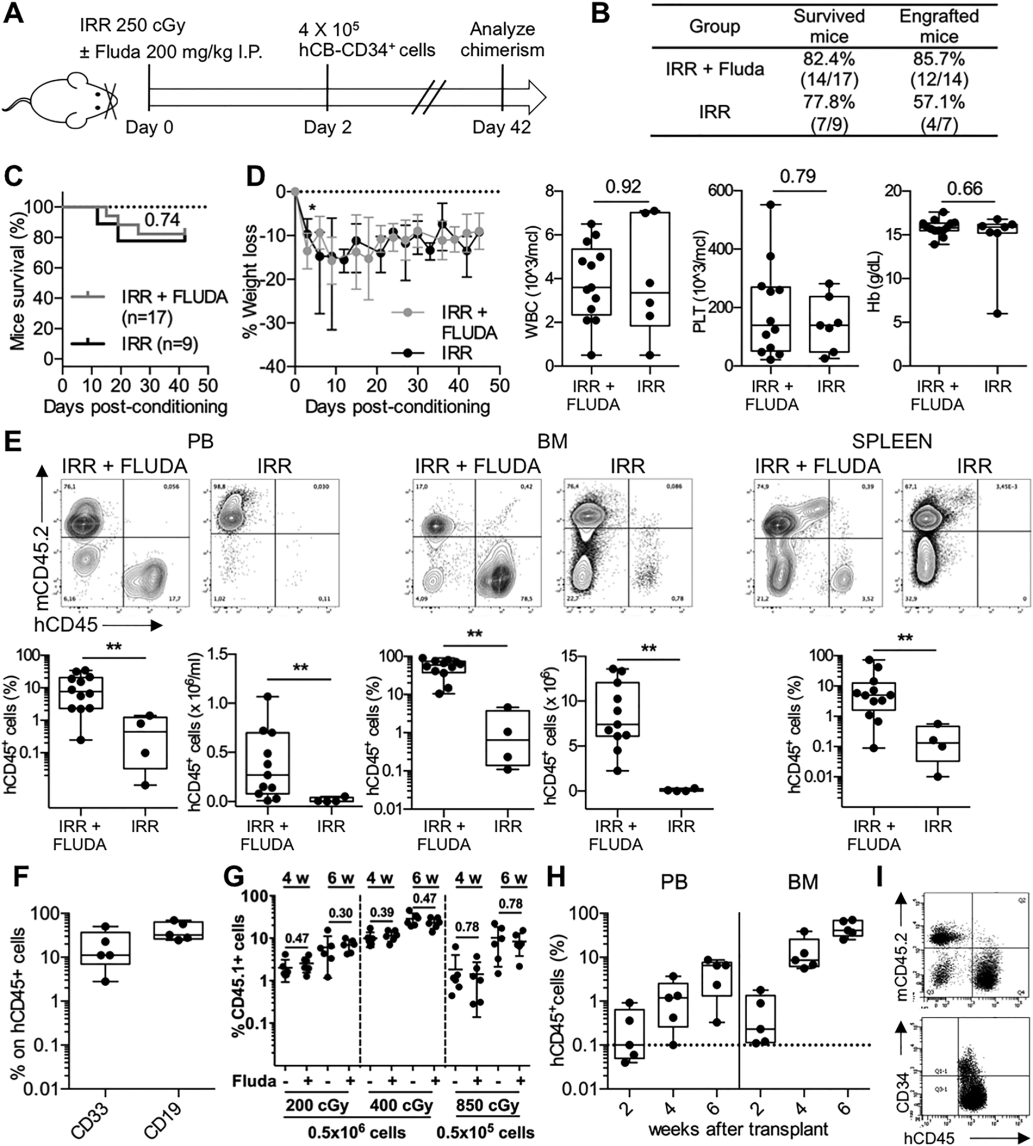
Fludarabine enables efficient human cell reconstitution. (**A**) Representation of experimental outline: irradiation (250 cGy) and 200 mg/kg fludarabine treatment of SCID/beige mice followed by transplantation of hCD34+ cells. Where not otherwise specified, mice were euthanized 6 weeks (day 42) after treatment to analyse chimerism in hematopoietic organs. Mice receiving only irradiation constitute the control group. (**B**) Percentages of engrafted mice that survived (defined by more than 0.1% hCD45 cells within BM) in the two experimental groups at the fixed end-point. 10 independent experiments (2–4 mice/experiment); (**C**) Kaplan-Meier curve of overall survival of mice treated with irradiation + fludarabine (gray line) *versus* irradiation (black line). *P* value by log-rank Mantel-Cox test. 10 independent experiments (2–4 mice/experiment); (**D**) SCID/beige mice receiving irr+ fluda or irr were weighed over 6 weeks following the conditioning procedure (on left) and their blood was collected at sacrifice for testing white blood cells (WBC), platelets (PLT) and hemoglobin (Hb) (on right). Percent change in body weight is represented as mean and standard deviation. Blood parameters are represented by boxplot graph, showing the exact data values by black dots. P values by Wilcoxon test. 10 independent experiments (2–4 mice/experiment); (**E**) Human engraftment in irr+ fluda treated or irr SCID/beige mice. On the top, representative dot plots showing hCD45+ cells in PB, BM and spleen 6 weeks after transplantation. On the bottom, proportion and absolute numbers of hCD45+ cells in the same groups. 10 independent experiments (2–4 mice/experiment); (**F**) Relative proportion of myeloid (CD33^+^) and B lymphoid (CD19^+^) cells within the human graft in irr+ fluda mice at 6 weeks. 2 independent experiments (2–3 mice/experiment); (**G**) Frequency of donor hematopoiesis in PB 4 and 6 weeks after transplantation of mice irradiated (200, 400 or 850 cGy), treated or not with fludarabine and injected with 0.5 or 0.05 × 10^6^ BM cells of congenic mice. 2 independent experiments (3 mice/group in each experiment); (**H**) Levels of human chimerism analysed in PB and BM of recipient mice at 2, 4 and 6 weeks after conditioning with irr+ fluda. 2 independent experiments (2–3 mice/experiment); (**I**) Long term human engraftment (at 12 weeks) in irr+ fluda treated mice and presence of hCD34+ precursors.

We decided to adopt the SCID/beige mouse model based on the fact that if mice are conditioned with a sublethal dose of irradiation, they exhibit low levels of human engraftment^15^. Fludarabine was injected intraperitoneally in mice that were previously irradiated with 250 cGy. Two days later, human cord blood (CB)-derived CD34^+^ cells (hCD34^+^) were injected intravenously (Fig. 1A). Mice irradiated with the same dose and transplanted with hCD34^+^ cells derived from the same CB donor but not receiving fludarabine were used as controls.

We determined the toxicities of these two conditioning regimens by measuring survival, body weight and the blood counts. The addition of fludarabine did not significantly worsen the survival rate of the treated mice over 6 weeks compared to control mice (p = 0.74), but caused a substantial reduction in body weight only at early points after conditioning. Otherwise, all blood parameters were comparable (Fig. 1C,D).

The addition of fludarabine to irradiation resulted in a larger extent of human engraftment, when compared to the control group receiving only irradiation (Fig. 1B). Quantification of human CD45^+^ (hCD45^+^) cells in BM at 6 weeks after the transplant revealed a significant difference between the two groups both in proportion (median 58.3% in Irr+ Fluda, range from 10.49 to 90.34%; 0.64% in Irr, range from 0.11 to 4.60%; p = 0.0011) and absolute numbers (median 7.4 × 10^6^ in Irr+ Fluda, range from 2.25 to 13.6 × 10^6^; 0.06 × 10^6^ in Irr, range from 0.01 to 0.30 × 10^6^; p = 0.0015) (Fig. 1E). A similar difference in the levels of human cell engraftment was also observed in spleen and peripheral blood (PB), in which in the presence of fludarabine the increase was 17- and 96-fold higher, respectively. BM analysis showed human engraftment to be multilineage, consisting not only of myeloid (CD33^+^) but also B lymphoid (CD19^+^) cells (Fig. 1F). Fludarabine by itself was not sufficient to increase human engraftment, resulting in engraftment levels significantly lower than those obtained with the combination of fludarabine and irradiation (data not shown).

In order to understand whether the synergistic effect of fludarabine was the result of remodelling the hematopoietic niche^10,11^ or an immunosuppressive effect, we tested the approach in a syngeneic murine transplantation model, using donor-recipient pair congenic for a CD45 polymorphism (Fig. 1G). The addition of fludarabine did not change the engraftment levels obtained with irradiation only both at 4 and 6 weeks post-transplantation, also after administrating very few donor cells, thus suggesting that the fludarabine effect should be related to its immunosuppressive effect rather than an activity on the BM microenvironment. Moreover, we proved that the capacity of murine splenocytes to proliferate after *in vitro* stimulation with Concanavalin A was markedly inhibited by fludarabine (Supplementary Fig. S1).

Notably, the administration of fludarabine promoted an early engraftment of human hematopoiesis in transplanted mice, in which it was possible to observe a detectable percentage of human cells in BM since the second week after transplant (median 0.23% hCD45^+^ cells, range from 0.11 to 1.78%) (Fig. 1H). The long-term reconstitution induced by fludarabine was durable, since we could still detect the presence of hematopoietic precursors CD34^+^ 12 weeks after transplantation (Fig. 1I).

### Fludarabine promotes AML engraftment

**Figure 2:**
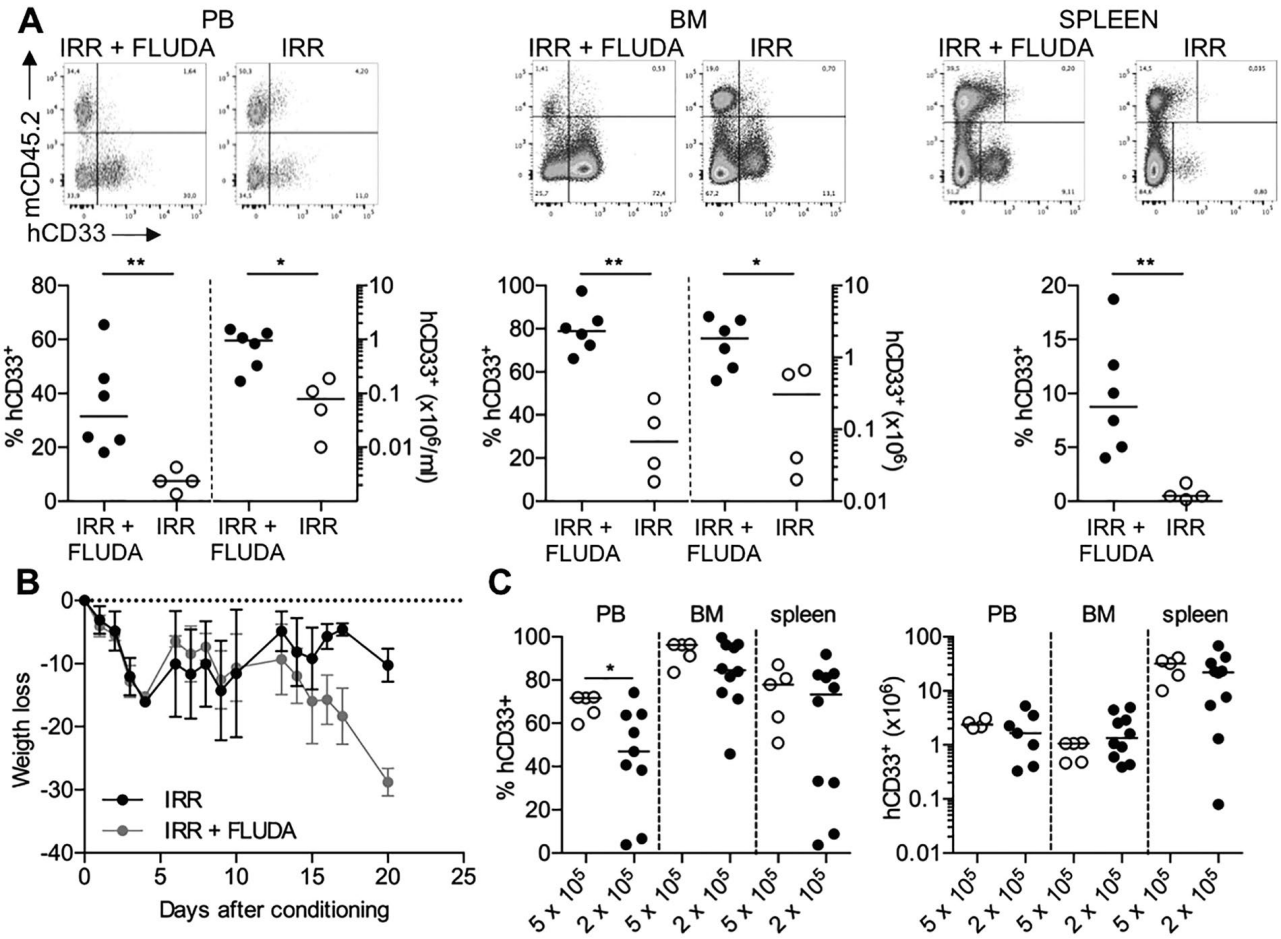
Fludarabine promotes AML cell line engraftment. (**A**) SCID/beige mice were intravenously injected with 2 × 10^6^ cells from the established human myeloid cell line KG-1 on day 2 post conditioning (irr+ fluda or irr). Mice were euthanized for the engraftment assessment in PB, BM and spleen when they developed signs of overt leukemia. Upper panels, exemplary data from one representative experiment; lower panels, presence (as percentages and numbers) of hCD33+ cells in PB, BM and spleen of transplanted mice. 2 independent experiments (2–3 mice/group in each experiment); (**B**) Weight loss in SCID/beige mice conditioned with irr+ fluda (gray line) or irr (black line) and transplanted with KG-1 cells. 2 independent experiments (2–3 mice/group in each experiment); (**C**) SCID/beige mice were treated with irr+ fluda and injected with two different doses (5 × 10^5^ or 2 × 10^5^ cells/mouse) of KG-1 cells. At euthanasia, the engraftment level in PB, BM and spleen was assessed by flow cytometry as a proportion (on left) or as absolute numbers (on right). 3 independent experiments (3–6 mice/group in each experiment).

**Figure 3:**
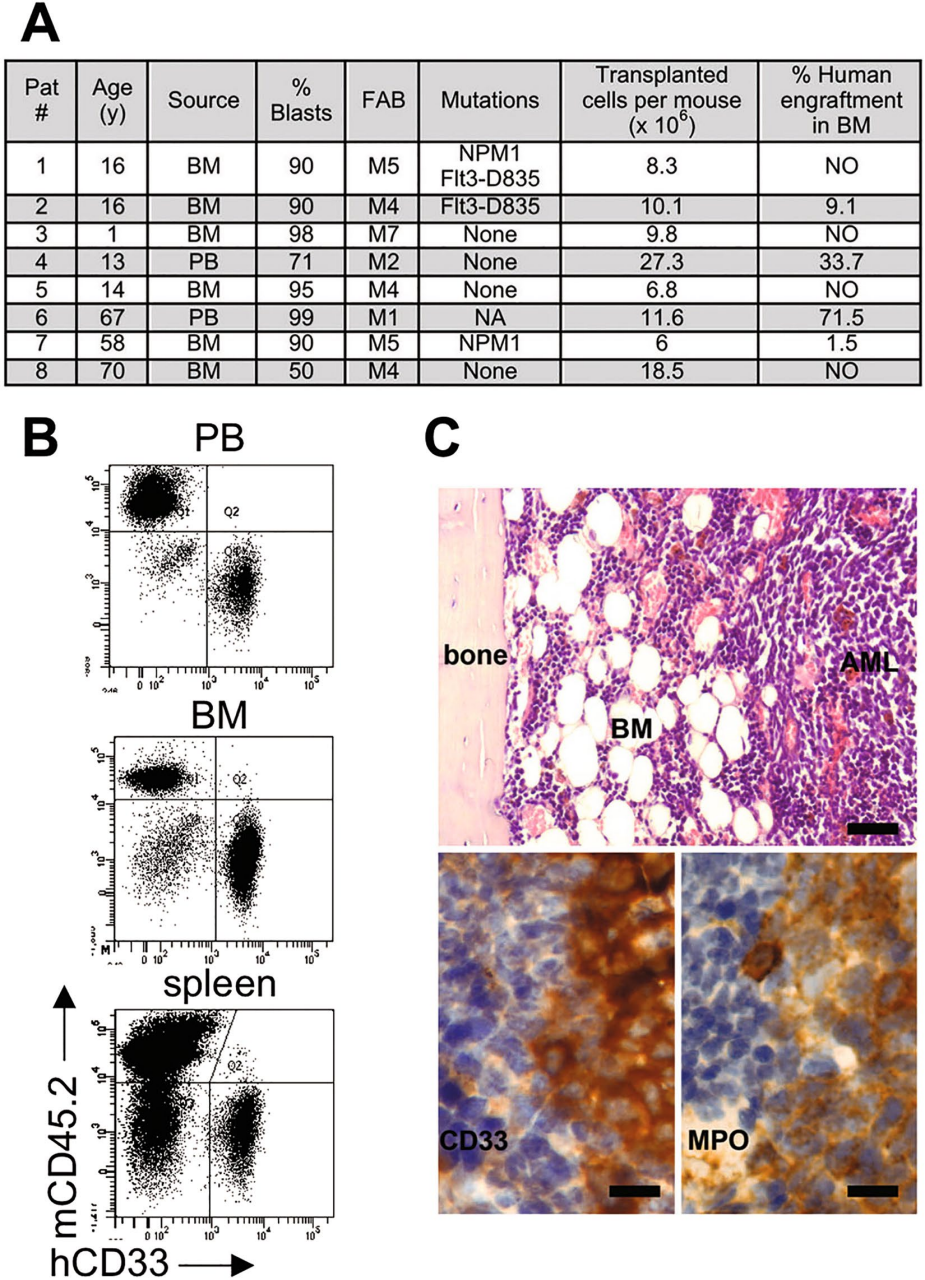
Fludarabine permits engraftment of primary AML blasts. (**A**) Clinical characteristics of AML patients (age, source and percentage of blasts, Fab classification, NMP-1 and Flt3 mutational status) and engraftment details (transplanted cell dosage and % human engraftment detected in BM at final analysis) following transplantation in SCID/beige mice conditioned with irr+ fluda. NA, not analysed. 8 independent experiments; (**B**) Representative dot plots showing hCD33^+^ blasts in PB, BM and spleen of SCID/beige mice previously treated with irr+ fluda (patient #6). (**C**) Representative paraffin sections from the same mouse (patient #6) stained with Hematoxylin and Eosin (top panel) to show the human blasts infiltrating the BM. The infiltrating blasts display a myeloid phenotype as proved by their immunoreactivity with anti-hCD33 and MPO antisera. Bars: 100 µm in the top panel and 20 µm in the bottom panels.

We applied the conditioning regimen to evaluate the effect on the engraftment of acute myeloid leukemia (AML) that is notoriously difficult to engraft in SCID models. Also in this case, the addition of fludarabine to irradiation could favour the engraftment of the AML cell line KG-1. The presence of high percentages of AML cells within BM (median 78.91%, range from 66.24 to 97.59%) was accompanied by signs of distress, including weakness, weight loss, hunched back, loss of ambulation, laboured breathing and paralysis, and mice were humanely killed within 3 weeks when transplanted with 2 × 10^6^ KG-1/mouse (Fig. 2A,B). Using the same conditioning strategy, it was possible to transplant lower numbers of cells (5 × 10^5^ or 2 × 10^5^/mouse) obtaining dose-dependent sustained engraftment levels, with a longer overall survival (from 21 to 29 days) (Fig. 2C). Finally, we evaluated if this preconditioning strategy could sustain the engraftment of primary AML blasts derived from 8 patients with various genetic backgrounds, including NPM-1 mutated, Flt3 mutated, NPM-1/Flt3 mutated and wild type (Fig. 3A). Strikingly, primary AML samples injected in fludarabine-preconditioned mice produced a detectable engraftment in 50% of transplanted AML cases, within the first 8 weeks after transplantation. Leukemic cells infiltrated BM (range from 1.5 to 71.5% hCD45^+^hCD33^+^, evaluated at 8 weeks) and hematopoietic organs (spleen, range from 0.8 to 7.6% hCD45^+^hCD33^+^ and PB, range from 1.4 to 19.6% hCD45^+^hCD33^+^, evaluated at 14 weeks), the primary sites of clinical AML (Fig. 3B,C). Flow cytometry data correlated with immunohistochemistry analysis (Fig. 3C).

## Discussion

In recent years, fludarabine has been adopted as a single agent or in combination with other drugs for the treatment of various hemato-oncologic malignancies and has been shown to be particularly effective in indolent lymphoproliferative disorders, predominantly chronic lymphocytic leukemia (CLL) and follicular lymphoma. Furthermore, combination regimens containing fludarabine have also been administered in the treatment of aggressive lymphomas and acute leukemias. The inclusion of fludarabine in the conditioning regimen of allogeneic stem cell transplantation was initially proposed for its remarkable immunosuppressive activities^8^. Most recently, due to its synergistic cytotoxic activity against both myeloid and lymphoid malignancies when used in combination with alkylating agents or radiotherapy, it has now become a standard of treatment in allogeneic stem cell transplantation^4–7^. Indeed, the use of fludarabine-containing regimens has modified the incidence and the degree of graft-versus-host disease (GVHD) in these patients.

In experimental BM transplantation, fludarabine has been mainly administered in graft-versus-host disease^12–14^. To date, no study has addressed the ability of fludarabine to favour the engraftment of normal and malignant hematopoiesis in a mouse model. Our data convincingly show that the administration of fludarabine in irradiated mice renders SCID/beige mice excellent recipients not only for normal human hematopoietic cells, but also for the leukemic counterparts.

The *in vivo* kinetics of fludarabine suggest to wait at least 48 hours before infusing hematopoietic stem cells to enable the complete clearance of the drug and potential toxic effects on the graft^3^.

Therefore, in our hands fludarabine can be exploited to obtain a fast and durable human engraftment in low-care immunodeficient strains. The mechanism of this phenomenon could be ascribed to the fludarabine-induced immunosuppression associated with the cytotoxic potential against lymphocytes due to the inhibition of STAT1 signaling^9^. The fact that fludarabine does not enhance the engraftment level in a congenic model seems to support this hypothesis. The inhibition of mitogen-induced murine splenocyte proliferation by fludarabine supports the hypothesis that it may control the host lymphocyte leakage that interferes with the human graft^16^. SCID/beige mice are congenic mice that possess both genetic autosomal recessive mutations SCID (Prkdc^scid^) and beige (Lyst^bg^). The beige mutation results in defective NK cells. The SCID mutation results in deficiency in V(D)J recombination, producing severe lymphopenia but not absolute absence of T and B cells. Indeed, through the incomplete penetrance (“leakiness”) of the scid mutation, occasional productive VDJ rearrangement can occur and give rise to clonal expansion of limited T and B cell clones^16^. A similar leaky phenotype is also described in SCID/beige mice^17^. Leaky SCID lymphocytes can respond to mitogens, are capable of producing cytokines and serum Ig, and may develop reaction to allogeneic tissue^18^. For instance, it is possible that the immunosuppressant drug fludarabine could affect the residual T and B lymphocytes in SCID/beige mice, that may represent a potential interference with graft acceptance.

In summary, we demonstrate that the administration of fludarabine promotes in SCID/beige mice an extensive and durable engraftment of normal human hematopoiesis that exceeds the levels currently achievable in other models. The engraftment comprised hematopoietic stem cells as well as myeloid and lymphoid cells and could be reproduced using cells sourced from myeloid malignancy. The disease phenotype observed in leukemia-engrafted mice recapitulated the features of the human counterpart. We conclude that fludarabine treatment may provide a tool to maximise normal and malignant xenotransplantation in otherwise inefficient but relatively inexpensive immunocompromised recipients such as SCID/beige mice. The approach could be applied to other existing models of diseases (e.g., SCID models of rare genetic disorders) that remain in need of sufficiently informative levels of human engraftment. Overall, our results provide novel and affordable information for the engraftment of human normal and malignant hematopoiesis in immunodeficient mice.

## Methods

### Study approval

All animal experiments were performed under license approved by the Italian Ministry of Health and in accordance with Italian Cancer Research guidelines. The use of umbilical cord blood (UCB) and AML samples was approved by the Ethics Committee of San Gerando Hospital-Monza and carried out in accordance with the Declaration of Helsinki. All samples were only processed from patients who had consented the use of biological material for ethically approved research.

### Cell lines and primary AML samples

Human AML cell line KG-1 (obtained from the ATCC) was maintained in culture, splitting every 2–3 days, in Advanced RPMI medium (Invitrogen) supplemented with 10% heat-inactivated fetal bovine serum (Biosera), 2 mM L-glutamine, 50 IU/ml of penicillin and 50 μg/ml of streptomycin (Lonza).

For primary acute myeloid leukemia samples, PB or BM samples from adult or pediatric patients were collected at diagnosis. Samples were enriched for mononuclear cells by using a Ficoll-Paque gradient, and subsequently frozen in 10% dimethyl sulfoxide solution (Sigma-Aldrich). Details of patients’ samples are provided in Fig. 3.

### Xenotransplantation procedures

Animals were used in accordance with a protocol approved by the Italian Ministry of Health. Adult (10–12 weeks old) SCID/beige (CB17.Cg-Prkdc^scid^Lyst^bg-J^/Crl) mice purchased from Charles River Laboratories (Calco, Italy) were sublethally irradiated (250 cGy) and treated with 200 mg/kg Fludarabine (Teva) by intraperitoneal administration, 48 h before intravenous injection of human cells.

For normal reconstitution, CD34^+^ progenitors from human CB were obtained by Ficoll-Paque Plus (GE Healthcare Europe) separation of the mononuclear fraction followed by immunomagnetic selection using the CD34 MicroBeads kit (Miltenyi Biotec). 10 independent hCD34^+^ batches were used for transplantation experiments, with an average purity of 81% (range: 70–95.2%).

For leukemic reconstitution, cultured AML cell line KG-1 or freshly thawed PB/BM samples from patients with AML were used for transplants.

Daily monitoring of mice for symptoms of disease (ruffled coat, hunched back, weakness and reduced motility) determined the time of euthanasia for injected animals with signs of distress.

### Engraftment evaluation

Human engraftment (defined as more than 0.1% human cells in murine BM) was assessed in PB, BM and spleen at defined time points (in the case of normal hematopoietic cells transplant) or at signs of distress (in the case of AML cell line KG-1 transplant). Engraftment of human cells (in the case of primary patient-derived AML cells) was evaluated in BM at 8 weeks after transplantation (analyzing femoral BM aspirates) and in spleen and PB at the time of euthanasia (14 weeks). Kaplan-Meier survival analysis and body weight assessment were performed on all animals.

For engraftment evaluation, 50 µL of PB were collected in heparin by tail bleeding, analyzed with hematology counter (Coulter AcT Diff, Beckman Coulter), and lysed with ACK (Ammonium-Chloride-Potassium) lysing buffer (Stem Cell Technologies). BM (mixed from tibiae and femora) was collected by flushing long bones, while splenocytes were collected by smashing spleen on a 70 µm cell strainer (Greiner Bio-One). Single cells suspensions were counted in Bürker chamber with Turk solution and processed for analysis by flow cytometry.

### Flow cytometry and histopathology

For flow cytometry analyses, fluorescent antibodies against murine CD45.2 (clone 104, eBiosciences) and against human CD45 (clone HI30), CD33 (clone P67.6), CD34 (clone 8G12), CD19 (clone SJ25C1) (BD Biosciences), and CD45 (clone HI30, Invitrogen) were used. The analyses were performed on a FACS CantoII (BD Biosciences).

Humeri of fludarabine-treated SCID/beige mice transplanted with primary human AML blasts were fixed in 4% formaldehyde in phosphate buffer, decalcified in 10% EDTA (Sigma-Aldrich) and routinely processed for paraffin embedding. Serial 5 µm-thick sections were stained with Hematoxylin-Eosin and stained with anti-human CD33 (# NCL-L-CD33, clone PWS44, 1:100; Novocastra™) and Myeloperoxidase (#NCL-MYELO, clone 59A5, 1:100, Novocastra™) antisera.

### Murine BM transplantation

For murine BM transplantation experiments, 12-week-old C57BL/6-CD45.2 mice were conditioned by irradiation alone or followed by injection of fludarabine and BM cells from congenic C57BL/6-CD45.1 mice were transplanted by a single intravenous injection within 48 hours from conditioning. In one experimental setting mice received a sublethal irradiation (200 or 400 cGy) with the infusion of 0.5 × 10^6^ donor cells/mouse and in the other one an irradiation of 850 cGy with 0.05 × 10^6^ donor cells/mouse.

For engraftment evaluation, 50 µL of PB were collected 4 and 6 weeks after transplant in heparin by tail bleeding, lysed with ACK buffer and stained with antibodies against murine CD45.1 (clone A20, eBiosciences) and CD45.2 (clone 104, eBiosciences).

### Immunosuppressive assay

5 × 10^5^ C57BL/6 murine splenocytes were labelled with PE-Cell Tracker (ThermoFisher Scientific), plated in the presence or in the absence of fludarabine (2.5 mcg/ml) for 24 hours, and then stimulated with ConA (3 mcg/ml). Controls consisted of splenocytes plated without ConA. Proliferation was assessed by flow cytometry after 72 hours. Cell counts were determined by adding CountBright absolute counting beads (Molecular Probe) to the flow cytometric samples.

### Statistical analysis

Unless otherwise stated, data are represented as median and range. Nonparametric Wilcoxon test for equality of the medians was used to calculate P-values. Significance is represented as follows: ^*^P < 0.05, ^**^P < 0.01, ^***^P < 0.001.

## Electronic supplementary material

Supplementary Figure S1

## References

[1] Goyama S, Wunderlich M, Mulloy JC (2015). Xenograft models for normal and malignant stem cells. Blood.

[2] McCormack E, Bruserud O, Gjertsen BT (2005). Animal models of acute myelogenous leukaemia – development, application and future perspectives. Leukemia.

[3] Aversa F (1998). Treatment of high-risk acute leukemia with T-cell-depleted stem cells from related donors with one fully mismatched HLA haplotype. N. Engl. J. Med..

[4] Andersson BS (2008). Once daily i.v. busulfan and fludarabine (i.v. Bu-Flu) compares favorably with i.v. busulfan and cyclophosphamide (i.v. BuCy2) as pretransplant conditioning therapy in AML/MDS. Biol Blood Marrow Transpl..

[5] Niederwieser D (2003). Low-dose total body irradiation (TBI) and fludarabine followed by hematopoietic cell transplantation (HCT) from HLA-matched or mismatched unrelated donors and postgrafting immunosuppression with cyclosporine and mycophenolate mofetil (MMF) can induce durable complete chimerism and sustained remissions in patients with hematological diseases. Blood.

[6] Rambaldi A (2015). Busulfan plus cyclophosphamide versus busulfan plus fludarabine as a preparative regimen for allogeneic haemopoietic stem-cell transplantation in patients with acute myeloid leukaemia: an open-label, multicentre, randomised, phase 3 trial. Lancet Oncol..

[7] Khouri IF (2001). Nonablative allogeneic hematopoietic transplantation as adoptive immunotherapy for indolent lymphoma: low incidence of toxicity, acute graft-versus-host disease, and treatment-related mortality. Blood.

[8] Terenzi A (1996). Efficacy of fludarabine as an immunosuppressor for bone marrow transplantation conditioning: preliminary results. Transplant. Proc..

[9] Frank DA, Mahajan S, Ritz J (1999). Fludarabine-induced immunosuppression is associated with inhibition of STAT1 signaling. Nat. Med..

[10] Tajima K (2010). Inhibition of STAT1 accelerates bone fracture healing. J. Orthop. Res..

[11] Furukawa M (2009). IL-27 abrogates receptor activator of NF-kappa B ligand-mediated osteoclastogenesis of human granulocyte-macrophage colony-forming unit cells through STAT1-dependent inhibition of c-Fos. J. Immunol..

[12] Giver CR (2003). *Ex vivo* fludarabine exposure inhibits graft-versus-host activity of allogeneic T cells while preserving graft-versus-leukemia effects. Biol. Blood Marrow Transplant..

[13] Weiss L, Abdul-Hai A, Or R, Amir G, Polliack A (2003). Fludarabine in combination with cyclophosphamide decreases incidence of GVHD and maintains effective graft-versus-leukemia effect after allogeneic stem cell transplantation in murine lymphocytic leukemia. Bone Marrow Transplant..

[14] Luznik L, Jalla S, Engstrom LW, Lannone R, Fuchs EJ (2001). Durable engraftment of major histocompatibility complex-incompatible cells after nonmyeloablative conditioning with fludarabine, low-dose total body irradiation, and posttransplantation cyclophosphamide. Blood.

[15] Kirkiles-Smith NC (2009). Development of a humanized mouse model to study the role of macrophages in allograft injury. Transplantation.

[16] Carroll AM, Hardy RR, Bosma MJ (1989). Occurrence of mature B (IgM+, B220+) and T (CD3+) lymphocytes in scid mice. J. Immunol..

[17] Mosier DE, Stell KL, Gulizia RJ, Torbett BE, Gilmore GL (1993). Homozygous *scid/scid;beige/beige* mice have low levels of spontaneous or neonatal T cell-induced B cell generation. J. Exp. Med..

[18] Carroll AM, Bosma MJ (1988). Detection and Characterization of functional T cells in mice with severe combined immune deficiency. Eur. J. Immunol..

