# Supplementary material for: Fludarabine as a cost-effective adjuvant to enhance engraftment of human normal and malignant hematopoiesis in immunodeficient mice
Source: Sci Rep. 2018 Jun 14;8:9125. doi: 10.1038/s41598-018-27425-x (PMC6002385; doi:10.1038/s41598-018-27425-x)
Supplement: Supplementary file 1 — Supplementary Figure S1 [file 41598_2018_27425_MOESM1_ESM.pdf]

Fludarabine as a cost-effective adjuvant to enhance engraftment of human normal and malignant hematopoiesis in immunodeficient mice.

Pievani A[1,2], Michelozzi IM[1], Rambaldi B[1], Granata V[1], Corsi A[3], Dazzi F[2], Biondi A[1], Serafini M[1*].

Figure S1. Treatment of C57BL/6 murine splenocytes with fludarabine leads to inhibition of mitogen-induced proliferation.

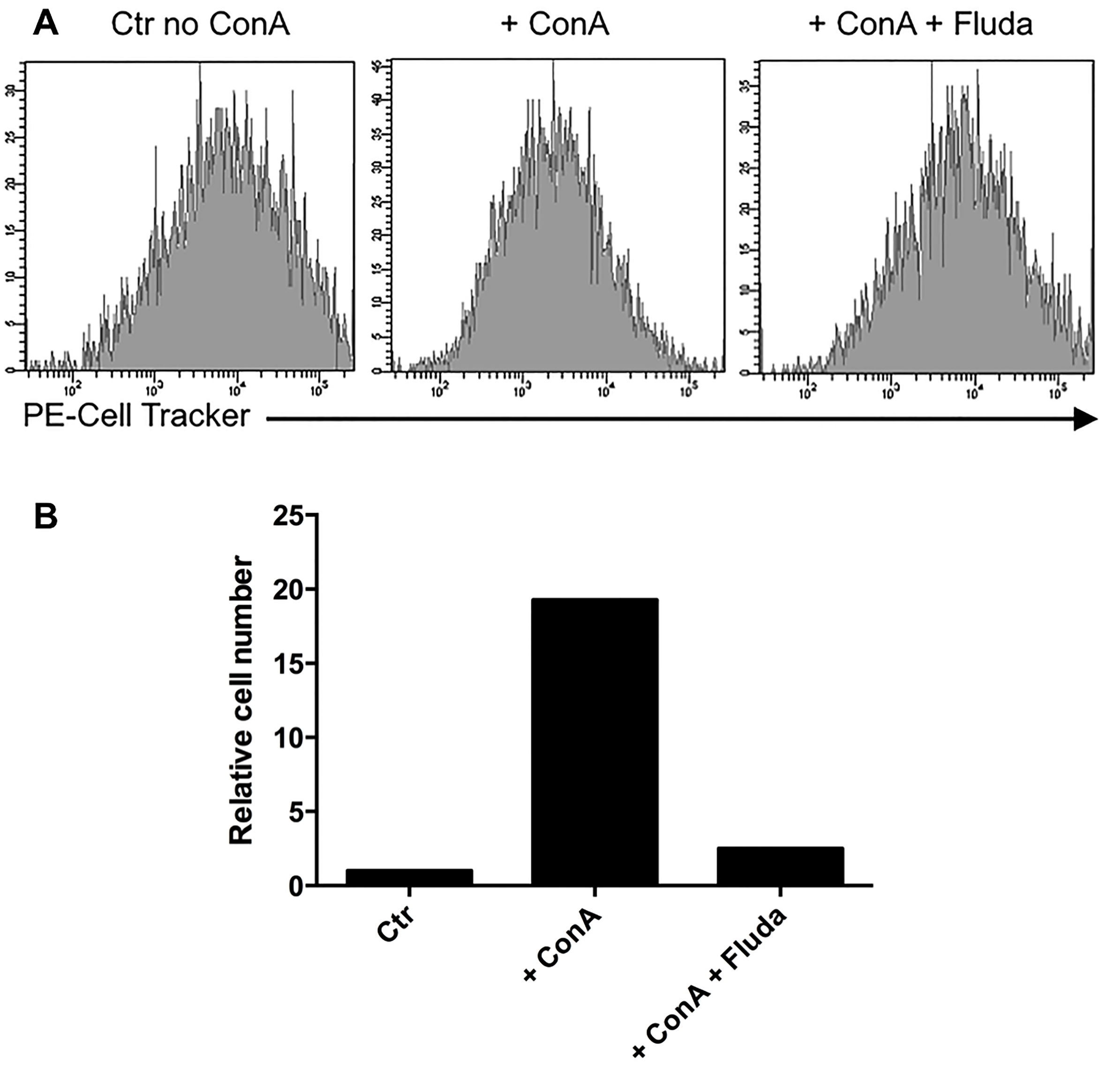

Splenocytes labelled with PE-Cell Tracker were treated or not with Fludarabine (2.5 mcg/ml) and cultured with or without Concanavalin A (ConA) for 72 hours. Proliferation and cells number were determined by flow-cytometry analyzing the Cell Tracker dilution and using the single-platform method. **A)** Representative hystogram plots were shown. **B)** Relative cell number was express in comparison to control (splenocytes not treated with Fludarabine and not stimulated with ConA).
